# Different PD-MCI criteria and risk of dementia in Parkinson’s disease: 4-year longitudinal study

**DOI:** 10.1038/npjparkd.2015.27

**Published:** 2016-01-14

**Authors:** Kyla-Louise Wood, Daniel J Myall, Leslie Livingston, Tracy R Melzer, Toni L Pitcher, Michael R MacAskill, Gert J Geurtsen, Tim J Anderson, John C Dalrymple-Alford

**Affiliations:** 1 Department of Psychology, University of Canterbury, Christchurch, New Zealand; 2 New Zealand Brain Research Institute, Christchurch, New Zealand; 3 Department of Medicine, University of Otago, Christchurch, New Zealand; 4 Brain Research New Zealand—Rangahau Roro Aotearoa, New Zealand; 5 Department of Medical Psychology, Academic Medical Center Amsterdam, Amsterdam, The Netherlands; 6 Department of Neurology, Christchurch Hospital, Christchurch, New Zealand

## Abstract

The Movement Disorder Society Task Force (MDS-TF) has proposed diagnostic criteria for mild cognitive impairment in Parkinson’s disease (PD-MCI). We hypothesized that the risk of dementia (PDD) varies across the different cutoff schemes allowed. A longitudinal study followed 121 non-demented PD patients for up to 4.5 years. In Part One, unique groups of patients were identified as PD-MCI at baseline using the MDS-TF requirement of two impaired cognitive test scores, with both scores classified as impaired at either (i) 2 s.d., (ii) 1.5 s.d. or (iii) 1 s.d. below normative data; relative risk (RR) of PDD was assessed at each criterion. In Part Two, the whole sample was reassessed and (i) RR of PDD determined when two impairments at 1.5 s.d. existed within a single cognitive domain, followed by (ii) RR of PDD in the unique group whose two impairments at 1.5 s.d. did not exist within a single domain (i.e., only across two domains). Twenty-one percent of patients converted to PDD. Part One showed that the 1.5 s.d. criterion at baseline is optimal to maximize progression to PDD over 4 years. Part Two, however, showed that the 1.5 s.d. cutoff produced a high RR of PDD only when two impairments were identified within a single cognitive domain (7.2, 95% confidence interval (CI)=3.4–16.6, *P*<0.0001; 51% converted). The RR when the 1.5 s.d. impairments occurred only across two different domains, was nonsignificant (1.7, CI=0.5–7.4, *P*=0.13; 11% converted) and similar to using a 1 s.d. criterion (1.9, CI=0.3–4.3, *P*=0.13; 8% converted). If the intent of a PD-MCI diagnosis is to detect increased risk of PDD in the next 4 years, optimal criteria should identify at least two impairments at 1.5 s.d. within a single cognitive domain.

## Introduction

In Parkinson’s disease (PD), cognitive impairment and other non-motor symptoms have a greater impact on quality of life than do motor symptoms and are associated with early mortality.^1,2^ Some 60% of patients may develop dementia (PDD) within 12 years of their motor symptoms and over 80% ultimately reach PDD, although an individual’s time course to dementia is highly variable.^3–5^ Risk factors include older age and severity of motor symptoms, but recognition of early cognitive impairment is particularly important.^6–10^ Recently, studies have examined the progression to PDD from mild cognitive impairment (PD-MCI). Although specific findings are variable, more PD-MCI patients than non-PD-MCI patients progress to PDD (19–62% vs. 0–20%, respectively) when followed 2 to 5 years after showing PD-MCI.^11–13^ In a cohort that was followed for 16 years, 91% of PD-MCI patients reached PDD, over four times that of the non-PD-MCI patients.^14^ It is difficult to compare these studies, however, as each used different diagnostic criteria to define PD-MCI.

The heterogeneous methods to ascertain PD-MCI has led to substantial variation in the percentage of patients classified as PD-MCI.^11,15–18^ To address this situation, standardized diagnostic criteria were proposed by the Movement Disorder Society PD-MCI Task Force (MDS-TF).^19^ The Task Force’s Level I criteria permit the use of global cognition scales or an abbreviated neuropsychological assessment that includes fewer than two tests in each of five cognitive domains (attention/working memory; executive function; episodic memory; visuoperceptual/visuospatial function; language) or when fewer than five cognitive domains are assessed. More specific, comprehensive (Level II) criteria require the use of more than one test in each of the cognitive domains and PD-MCI is confirmed when any two (or more) impaired neuropsychological test scores are present but everyday function is generally preserved. Nonetheless, the Level II recommendations still permit several alternative criteria, for example, cutoffs ranging 1–2 s.d. below normative data to define an impaired score.^19^

We hypothesized that the risk of progression to PDD differs across three common cutoff variants for PD-MCI (1, 1.5 and 2 s.d.) permitted by the MDS-TF recommendations. Moreover, the high probability of eventual PDD for all patients means that the validity of PD-MCI criteria should be determined by whether they detect patients at increased risk of PDD within a defined period of time. Here, we specified a 4-year window as a suitable period of time because we considered this relevant for the use of PD-MCI in both clinical practice and potential therapeutic interventions.^20^ There is, however, a second important issue that has also not been addressed: does the distribution of impaired scores across domains influence the risk of conversion to dementia? On the basis of evidence from the non-PD literature on MCI,^21,22^ we hypothesized that having a minimum of two impaired test scores within a single cognitive domain would result in a higher risk of PDD compared with having a minimum of two impaired test scores spread across different cognitive domains.

## Results

### Participants

The mean patient age at baseline was 66 years (s.d.=8 years). Table 1 summarizes the demographics and neuropsychological data of the patients and healthy control group at baseline. There was a 21% conversion rate to dementia for the PD patients in the 4-year period (14 men and 11 women; 25 out of 121 patients). As expected, patients who later converted to PDD were older, had longer symptom duration and worse motor symptoms, neuropsychological and functional measures at baseline than patients who did not convert to PDD. At baseline, 26 out of the 121 non-demented patients experienced hallucinations. A greater proportion of converters (13 vs. 12; 52%) relative to non-converters (13 vs. 83; 14%) experienced hallucinations at baseline (chi-square=17.39, *P*<0.0001; Table 1). Fifteen of the 25 patients (60%) who converted to PDD experienced hallucinations at the time of conversion, whereas only 24 of the 96 non-converters experienced hallucination at their last assessment (chi-square=11.12, *P*<0.001). Relatively few patients (9 out of 121) received medication that may affect their cognitive performance during the study. At baseline, only one patient who did not convert to PDD at follow-up was on antipsychotic medication and one that did convert to PDD was receiving rivastigmine. Neither of these patients remained on these medications at their follow-up assessments. At follow-up, seven patients were on medication that could affect their cognition. Six of these seven patients were on antipsychotic medication (three of whom had progressed to PDD), and three were receiving donepezil (one of whom had progressed to PDD).

### Part one: comparison of progression to PDD from PD-MCI defined by three cutoffs

#### Risk of PDD

The first stage of analysis showed that the 46 patients classified as PD-MCI using the 2 s.d. cutoff had a significantly higher risk of progression to PDD than the remaining 75 PD patients (relative risk, RR=4.2, confidence interval (CI)=2.2–10.1, *P*<0.0001; Table 2). A similar finding was evident in the 10 patients missed by the 2 s.d. classification but defined as PD-MCI using the 1.5 s.d. cutoff (RR=4.9, CI=1.4–15.1, *P*=0.005). By contrast, the additional group of 40 patients classified as PD-MCI using the 1 s.d. cutoff criterion did not show a significantly greater risk of PDD progression than the remaining 25 patients not classified as PD-MCI under any of these three initial criteria (RR=1.9, CI=0.3–4.3, *P*=0.13).

#### Reversions from PD-MCI

Under each criterion, there were individuals who at their latest follow-up were classified as reverting to a non-PD-MCI status (i.e., to relatively ‘normal’ cognition under that criterion). For the 2 s.d. cutoff, 20% (9/46) of patients had reverted to a non-PD-MCI status at their last assessment (i.e., no longer having two impairments below 2 s.d.). Of these nine reverters, seven had scores that also remained below a 1.5 s.d. cutoff, but no longer met the 2 s.d. cutoff. There was just one reversion in the extra 10 patients identified as PD-MCI under the 1.5 s.d. criterion (i.e., in the group whose baseline scores reached the 1.5 s.d. cutoff but not the 2 s.d. cutoff). The reversion rate in the group of patients who met only the 1 s.d. criterion was 20% (8/40).

#### Healthy control group

Unlike the PD sample, none of the matched control group converted to dementia during the follow-up period. Only one control participant met the 2 s.d. criterion at baseline (showing one impairment within each of the two domains), two met the 1.5 s.d. criterion (each showing one impairment within each of the two domains) and 12 met the 1 s.d. criterion for MCI. By the end of the 4-year follow-up, however, the first control was no longer classified as MCI under the 2 s.d. criterion (having one impairment within each of the two domains, but now at 1.5 s.d.), while the two who had met the 1.5 s.d. criterion and six of those who had met the 1 s.d. criterion no longer met any MCI criterion.

### Part two: influence of the distribution of impaired scores within or across domains

The analysis in Part One led us to select the 1.5 s.d. cutoff as optimal for the second phase of analysis. The reason for this selection was that (i) the 1 s.d. criterion failed to identify an increased risk of progression to PDD, (ii) a similar RR of conversion to PDD was found for both the 2 s.d. and 1.5 s.d. criteria, (iii) the reversion rate was lower in the 1.5 s.d. criterion and (iv) combining the 2 s.d. and 1.5 s.d. groups captured a greater percent of converters to PDD. We therefore re-examined the entire sample of followed patients to look at the influence of the distribution of impaired scores at baseline (that is, using all the patients meeting a 1.5 s.d. criterion, including those meeting a 2 s.d. cutoff at baseline). The focus here was first to determine the relative risk of PDD when two (or more) impairments at 1.5 s.d. were present within a single cognitive domain. We then examined risk when only a single impairment at 1.5 s.d. was evident across at least two cognitive domains (that is, never two impairments within any one domain, but rather just one impairment at 1.5 s.d. in each of two or more domains). Of course, all of the PD-MCI patients classified using the 2 s.d. criterion by definition also met one of these 1.5 s.d. criteria.

#### PD-MCI at 1.5 s.d. with two impairments within a single domain

The group of 37 patients classified as PD-MCI at baseline using the criterion of two impairments at 1.5 s.d. in one cognitive domain (of whom 51% converted to PDD), had a high relative risk of progression to PDD compared with 84 patients (of whom 7% converted to PDD) who did not meet this criterion (RR=7.2, CI=3.4–16.6, *P*<0.0001; Table 2; 76% of all conversions to PDD). This risk was not influenced solely by the subgroup of patients (*n*=26; 54% converted to PDD) who showed two impairments at 2 s.d. within a single domain (RR=4.7, CI=2.4–8.7, *P*<0.0001), because the relative risk of the remaining subgroup of patients (*n*=11; 45% converted to PDD) with two impairments at 1.5 s.d. (but not 2 s.d.) in one domain was also high (RR=6.4, CI=2.2–15.6, *P*=0.0001).

#### PD-MCI at 1.5 s.d. with a maximum of one impairment in each of two domains

The additional group of 19 patients classified as PD-MCI using the criterion of only one impairment at 1.5 s.d. in each of two (or more) domains (of whom 11% converted to PDD) did not show a significantly increased risk of progressing to PDD compared with the remainder of PD patients (RR=1.7, CI=0.5–7.4, *P*=0.13).

#### Reversions from PD-MCI at 1.5 s.d

The proportion of patients reverting to a non-PD-MCI status was only 3% with the 1.5 s.d. criterion of two impairments in a single-domain, but was 16% with the 1.5 s.d. criterion of one impairment in two domains. Combined across these two 1.5 s.d. criteria, the reversion rate was 5% (that is, including scores below either the 2 s.d. or 1.5 s.d. cutoff; 3 of 56 patients).

#### Progression over time and pattern of domain impairments

**Figure 1:**
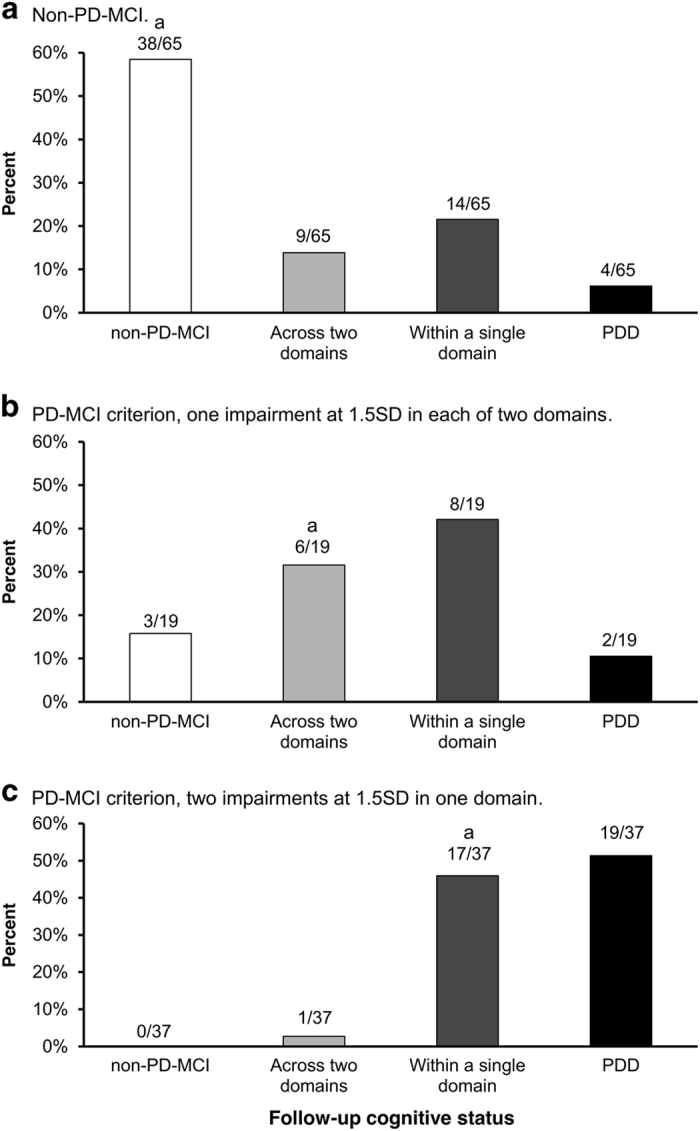
Follow-up cognitive status in the non-PD-MCI group at baseline (**a**) and the PD-MCI subgroups meeting the criterion of one impairment at 1.5 s.d. in each of two domains (**b**) and the criterion of two impairments at 1.5 s.d. in one domain (**c**). ^a^Original criterion at baseline. non-PD-MCI, patients not meeting the PD-MCI criterion; PD-MCI, Parkinson’s disease patients with mild cognitive impairment.

Support for the conclusion that two impairments within a single domain was effective in identifying PD-MCI patients at risk of PDD over a 4-year period was found when the progression of cognitive status was assessed in three groups of patients that were characterized differently at baseline using 1.5 s.d. criteria. More than half of the patients who did not meet either of the 1.5 s.d. criteria at baseline remained non-PD-MCI over the 4-year period (Figure 1a). It is pertinent that patients who met the PD-MCI criterion (at baseline) of a maximum of one impairment in each of two domains tended to progress to meet the alternative PD-MCI criterion of two impairments in one domain, rather than to PDD (Figure 1b). In contrast, those who met the PD-MCI criterion (at baseline) of two impairments in one domain tended either to remain stable at this criterion or progress to PDD (Figure 1c).

**Figure 2:**
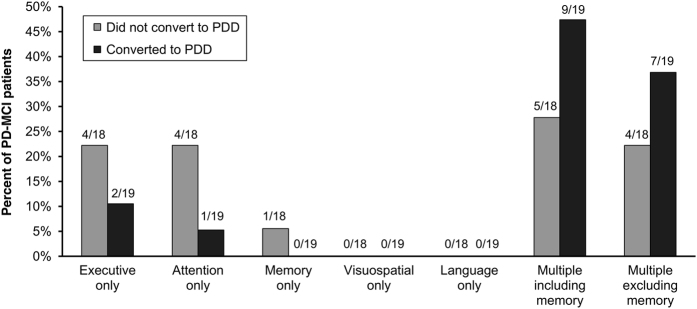
Pattern of domain impairments at baseline in PD patients meeting the criterion of at least two impairments at 1.5 s.d. in one domain. PDD, patients who met level II criteria for Parkinson’s disease with dementia; PD-MCI, Parkinson’s disease patients with mild cognitive impairment.

Figure 2 shows the pattern of domain impairments at baseline in the PD-MCI group who had at least two impairments at 1.5 s.d. within at least one of the five cognitive domains. Note that multiple-domain impairments therefore meant that they had two impairments in each domain of two or more domains. Most of these PD-MCI patients (25 of 37) had multiple-domain impairments (thus defined). Sixty-four percent of the patients with multiple-domain impairments converted to PDD (16 of 19), whereas only 25% of those with single-domain impairments (3 of 12) converted to PDD. All PD-MCI patients who had multiple-domain impairments that included memory, irrespective of conversion or not, had a mixture of other domains involved (memory and executive, *n*=5; memory and visuospatial, *n*=3; memory, executive and attention, *n*=4; memory, executive and visuospatial, *n*=1; memory, executive, attention and visuospatial, *n*=1). Multiple-domain impairments that did not include memory were: executive and attention, *n*=5; executive and visuospatial, *n*=3; and attention and visuospatial, *n*=3. No PD-MCI patients had single-domain impairments in visuospatial function or language. There was no obvious pattern to the domains impaired and conversion to PDD.

## Discussion

The diagnosis of PD-MCI is important in facilitating patient management, prognosis and especially opportunities for novel therapeutic interventions.^8–10,19,20^ The explicit intent of a PD-MCI diagnosis is to identify patients who are at risk of PDD, and this can only be determined by longitudinal follow-up of patients. Dementia is an outcome facing most PD patients as the disease unfolds and multiple brain networks become increasingly disrupted.^23–26^ The validity of a PD-MCI diagnosis is therefore most relevant in the context of the window of time when risk is evaluated. Our longitudinal study evaluated this risk over a 4-year period, a pertinent medium-term time-frame from the patient and their carer’s perspective.

In a typical age group of patients (42 to 80 years, mean of 66 years; mean disease duration of 6 years at the start of the study), we found that the 1 s.d. cutoff did not identify patients who were at higher risk of PDD over the next 4 years than patients who did not meet any PD-MCI criterion at baseline. By contrast, the criterion of impairments at 1.5 s.d. below normative data, when at least two such deficits occurred within one cognitive domain, has high validity for PDD risk in a 4-year period. This criterion classified a group with a 51% progression rate to PDD, the highest proportion of conversions for the criteria we examined. The literature on MCI in non-PD populations has established that poor performance on multiple tests within a domain is likely to identify worsening cognition over time.^21,22^ The alternative 1.5 s.d. criterion, which did not require two impairments within a single domain, was similar to the 1 s.d. criterion in that it did not identify patients with a significantly increased risk of PDD progression over a 4-year period. Indeed, this alternative 1.5 s.d. criterion instead may identify patients who were at an earlier stage of cognitive decline because many of these patients were more likely to convert to having two impairments within a single domain than to PDD. The option of 2 s.d. as a cutoff criterion has good concordance with a consensus clinical diagnosis of PD-MCI when neuropsychological data are available for assessment.^18^ However, the 2 s.d. criterion applied to our sample produced a lower relative risk estimate because it missed the additional conversions to PDD captured by the 1.5 s.d. cutoff, especially when two impairments occurred within one cognitive domain. The 2 s.d. criterion also produced an apparent higher rate of reversion to a non-PD-MCI status at follow-up than did the specific 1.5 s.d. criterion requiring two impaired scores within a single domain (20% vs. 3%, respectively). A recent proposal suggested the use of 2 s.d. cutoffs for PD-MCI criteria,^18,27^ but that study used a cross-sectional design and thus did not assess progression over time to PDD. The longitudinal design of the present study suggests instead that a 1.5 s.d. cutoff is sufficient to maximize the risk of progression to PDD.

PD-MCI criteria using different cutoff values result in a percentage of patients classified as impaired at a given point in time that can vary from as high as 92% to as low as 10%.^15–18^ The current consensus based on non-standardized PD-MCI criteria is that 25–30% of PD patients at any given time will demonstrate PD-MCI when the most common cutoff of 1.5 s.d. is administered, but even then significant variation is found across centers.^15^ Similarly, within our study, 46% of patients at baseline were PD-MCI when a 1.5 s.d. cutoff with only a single impairment in each of two cognitive domains was applied across the entire sample, but 31% were PD-MCI when the minimum of two impairments within a single domain was met. The proportions differed again if the entire sample was evaluated using 2 s.d. or 1 s.d. cutoffs (38% and 79%, respectively). Hence, different permissible MDS-TF cutoffs (1, 1.5 or 2 s.d.) contribute to considerable variation in the proportion of PD-MCI identified within a given sample of patients. More importantly, our 4-year study illustrates that different cutoff variants identify groups of patients with markedly different relative risks of progression to PDD, and different rates of reversion to non-PD-MCI.

Several factors could influence conversion or not to PDD within any group of patients. The small proportion of PD patients who do not progress to PDD until very old age may be protected by genetics and/or lifestyle factors that confer resilience to brain pathology.^28,29^ Some patients may be misdiagnosed as PD-MCI because either their depleted dopaminergic function or their treatment regimens may induce various fronto-striatal dysfunctions that cause cognitive impairment that does not necessarily portend progression to PDD.^30^ There are also contributions to risk from general factors such as age, presence of REM sleep disorder and additional neuropathology.^6,7,24,31,32^ In some patients, the extent of decline from premorbid level of intellectual function may be more relevant than rigid cutoffs.^33^ Hence, consideration of age or other variables may improve the predictive value of the specific criterion suggested by our study. Like other studies of conversion to PDD from PD-MCI, we found similar rates of conversion to PDD between the sexes when the 1.5 s.d. criterion was used (10 men and 9 women).^11,12^

The pattern of cognitive domain impairments observed in the patients who met the criterion of two impairments at 1.5 s.d. in a single domain showed no obvious association with conversion to PDD, which is consistent with the heterogeneity of failing cognition in PD.^11,13,34–36^ Like other studies, multiple-domain impairments may be more common in PD-MCI^18^ and more likely to be associated with conversion to dementia.^13^ The frequency of multiple-domain impairments will vary, however, with the specific PD-MCI criteria that are applied. In some studies, most PD-MCI patients showed multiple impairments, often as high as four domains impaired, when only a single impairment per domain was required.^18,33^ The present study had only one patient with four impaired domains, when using two impairments necessary for any domain to be considered deficient. Multiple deficit scores within a single domain, however, appears to be optimal to identify conversion to PDD within 4.5 years. Neither the language nor visuospatial domains showed single impairments using our criteria, so it is possible that worsening attention, executive function and memory may reflect the earlier signs of global cognitive decline in PD, similar to that reported by Pedersen *et al.* ^12^

The strength of the current study is its longitudinal design and the use of Level II assessments for both PD-MCI and PDD diagnoses. It evaluated for the first time the validity of different recommended PD-MCI criteria in relation to PDD progression. The use of a specified medium-term window for progression is highly relevant because the high rate of eventual progression PDD means that virtually all PD-MCI criteria will identify conversion to PDD if the follow-up period is sufficiently long. The 4-year window is particularly relevant for patients and their families, as it meets their expectations for clinical management while also allowing for the assessment of potential therapeutic outcomes.

One limitation to our study, like similar studies, is that the sample size was modest (*n*=121) and there was potential for bias owing to an attrition rate of 18%. Our sample size was twice that of three studies that examined PDD progression over 3–5 years (*n*=51–64),^11,13,14^ but smaller than one study that followed patients over 3 years (*n*=167).^12^ Our attrition rate (18%) was half that of two of these studies (41 and 48%),^13,14^ equivalent to one study (18%)^11^ and larger than another (8%).^12^ The current data will, however, contribute to an international consortium to enable better statistical precision for determining relative risk of PDD from different cutoff options, and the ability to assess the influence of potential modifiers such as age and education.^37^

A second limitation is that the identification of PD-MCI may depend on the number of tests we used per domain, and especially the sensitivity and psychometric qualities of our individual tests. The uneven number of tests per domain in our study could mean that the attention and executive functioning domains, each of which consisted of six tests, may be more likely to detect impairment than, say, the visuospatial domain for which four tests were used. However, Goldman *et al.* ^27^ found that having more than two tests in the attention and/or executive function domains did not increase the probability of detecting an impairment and that having more tests in memory, visuospatial functioning and language only increased by 5% the chance of finding more domain impairments. In terms of the sensitivity of tests, there is mixed evidence whether ‘frontal’ or ‘posterior’ cortical tests provide good predictors of decline to PDD.^11,30,34–36^ Indeed, the allocation of tests to cognitive domains may also differ across experts because tests vary in their domain purity. This issue is relevant to the current findings in which multiple impairments within a domain better identified those PD-MCI patients at risk of conversion to PDD, at least over a 4-year period. For example, executive function tests can vary in their domain allocation^12,18^ and visuospatial tests range from specific measures through to more complex tasks that also involve orientation, attention, memory and executive function.^38^ That is, the selection of tests and their domain purity may contribute to variation in the frequency of impairments reported across studies. These limitations emphasize the need for further evaluation of cognitive impairments and progression to PDD.

The conclusion from the current study is to adopt a specific PD-MCI criterion when the intent is to identify a medium-term (up to 4 years) risk of progression to PDD. For this purpose, the requirement of at least two deficits at 1.5 s.d. below normative data within any single cognitive domain provides a valid PD-MCI diagnosis that optimizes the relative risk of progression to dementia. Deciding upon the selection and number of tests required remains a major task facing the Parkinson’s disease research community.

## Materials and Methods

### Participants

**Figure 3:**
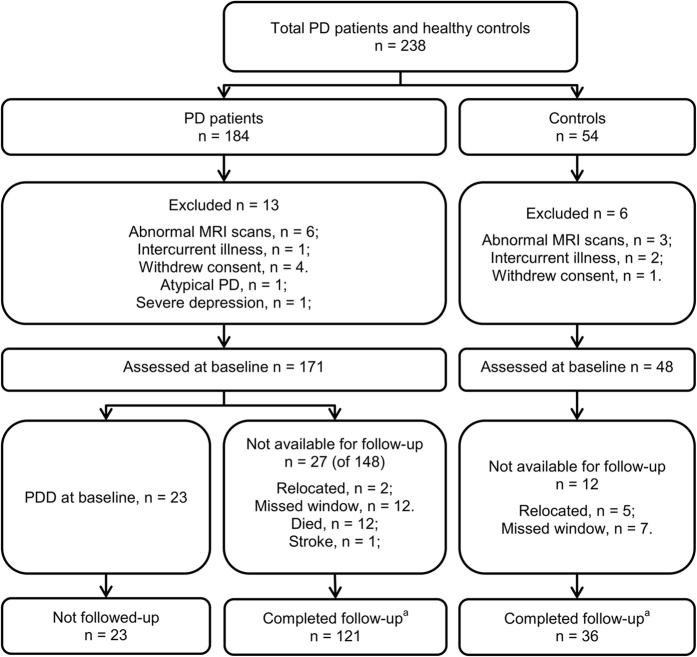
Participant recruitment, exclusions and total followed over 4 years. ^a^Assessments were conducted at baseline and every 1 to 2 years; patients with dementia were not followed further. MRI, magnetic resonance imaging; PD, Parkinson’s disease; PDD, Parkinson’s disease with dementia.

A convenience sample of 184 PD patients was recruited from our research institute and movement disorders clinic. Comparison of the rate of progression to PDD in this sample was made by recruiting 54 age-, sex- and education-similar healthy controls who volunteered in response to community advertisements and did not report subjective cognitive complaints. Figure 3 shows recruitment, exclusions and retention of participants,^39^ which resulted in a final followed-up sample of 121 PD patients, none of whom met PDD criteria^40^ at baseline, and 36 controls. Patients were diagnosed using the UK Parkinson’s Society criteria^41^ and had motor symptoms present for at least 1 year at study entry (mean symptom duration=6 years, s.d.=4 years) to minimize the inclusion of those with dementia with Lewy bodies. Atypical parkinsonian disorder, other medical conditions (e.g., history of moderate/severe head injury, stroke, early-life learning disability, major psychiatric or medical illness in the previous 6 months), poor English (precluding testing) were the exclusion criteria. Patients were also excluded at baseline if they had Parkinson’s disease with dementia.^40^ A PDD diagnosis at any point in the study required the presence of significant impairments (2 s.d. below normative data) in at least two of five cognitive domains, plus evidence of significant impairment in everyday functional activities, not attributed to motor impairments. Everyday function was assessed from interviews with a significant other, based on evidence obtained from the Reisberg IADL-scale, Clinical Dementia Rating and Global Deterioration Scale to attribute non-dementia status or PDD.^42,43^ Direct evidence from a significant other was not available, at baseline only, in 39 PD patients. However, we confirmed baseline non-dementia status in these cases from contemporaneous clinical notes and from detailed patient interview by an experienced examiner, followed by consensus discussion.

Comprehensive neuropsychological assessments fulfilling the MDS-TF Level II requirements for PD-MCI (for tests see below)^19^ were undertaken at study entry and subsequently every 1 to 2 years, for up to 3.5–4.5 years later (mean=46 months, s.d.=8 months). Progression to PDD before the end of the 4.5-year period was treated as an *a priori* end point, with no further follow-up. All the participants took their usual medications on the day of testing to allow optimal performance during the morning test sessions. The study was approved by a local ethics committee of the New Zealand Ministry of Health, with informed consent provided by all the participants.

### Neuropsychological assessment

Five cognitive domains were examined, with tests conducted over two sessions.^16,38,44^ Executive function was assessed using Stroop interference, letter fluency, category fluency and category switching (from the Delis-Kaplan Executive Function System^45^), and action fluency and Trails B. Attention, working memory and processing speed was evaluated using digits forwards/backwards, digit ordering, map search task (from the test of everyday attention), Stroop color reading, Stroop word reading and Trails A. Episodic memory was measured with the California Verbal Language Test-II Short Form (acquisition, short and long delays), and the Rey Complex Figure Test (short and long delays); impairment in either or both delay components of each memory test counted as one impairment. Visuoperceptual/visuospatial performance was determined using judgment of line orientation, fragmented letters test, the picture completion test and the Rey Complex Figure Test-Copy. Language was assessed using the Boston Naming Test, Dementia Rating Scale-2 similarities sub-test, and the language component of the Alzheimer’s Dementia Assessment Cognitive Scale (object and finger naming, commands, comprehension, spoken language and word-finding difficulties). Scoring of the neuropsychological tests was conducted using age- and education-adjusted normative data. Participants also completed the Montreal Cognitive Assessment.

### Application of PD-MCI criteria

There were two phases to the analysis. In Part One, we applied three commonly used and accepted PD-MCI cutoff criteria to examine their association with risk of progression to PDD, irrespective of whether the minimum of two impairments occurred within a cognitive domain or across different cognitive domains. In Part Two, we examined the additional issue of the distribution of impairments across domains, focusing on the optimal cutoff criterion resulting from the analysis in Part One. That is, we again determined risk of progression with a minimum requirement of two impairments, but first with both impairments within a single cognitive domain and then when there was only one impairment in each of two or more domains.

#### Part one

**Figure 4:**
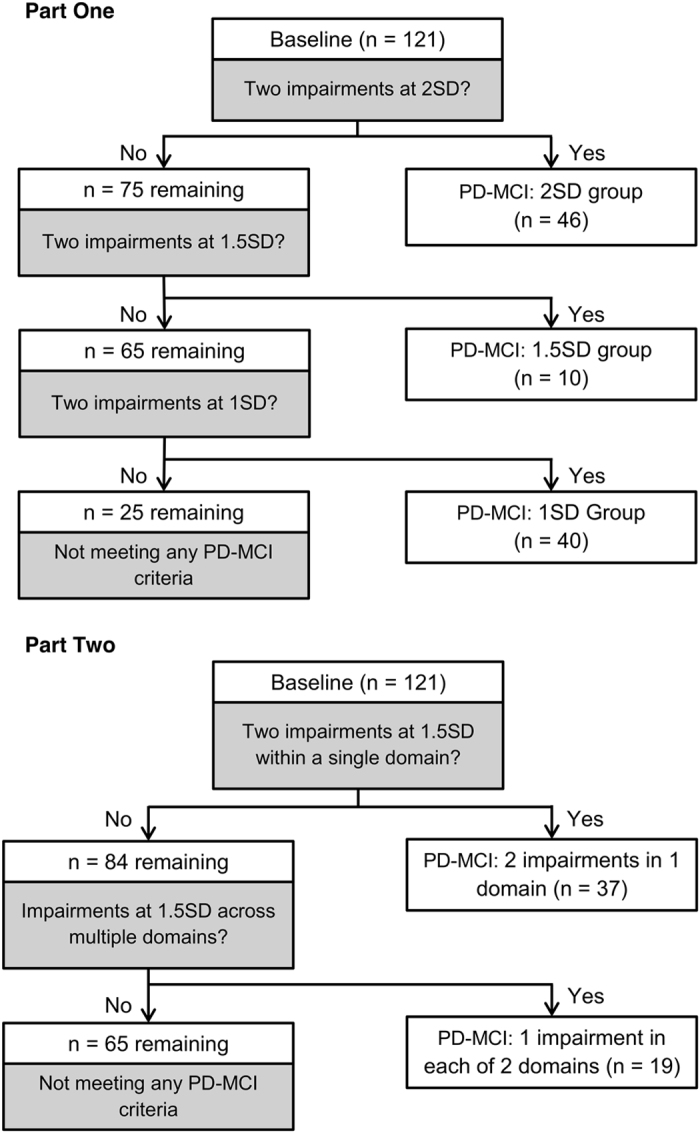
Allocation of non-dementing PD patients at baseline to different PD-MCI groups in Part One (using 2 s.d., 1.5 s.d. and 1 s.d. cutoff scores, respectively) and Part Two (alternative PD-MCI criteria for 1.5 s.d.). PD-MCI, Parkinson’s disease patients with mild cognitive impairment.

To test the unique contribution of each PD-MCI criterion, we generated three mutually exclusive groups of PD-MCI patients using a stepwise process. The three primary s.d. cutoff scores were applied sequentially to signify impairment on any test measure (Figure 4, Part One). First, we identified patients who had two scores that were 2 s.d. or more below normative data, then from the remainder we identified those with two scores that were 1.5 s.d. but better than 2 s.d. below normative data, and last those with two scores that were 1 s.d. but better than 1.5 s.d. below normative data. Thus each PD-MCI group in Part One consisted of an independent and discrete sample of patients for analysis purposes. In clinical practice, any patient who met a 2 s.d. criterion would also by definition meet the 1.5 s.d. or 1 s.d. criterion (and similarly those meeting 1.5 s.d. also exceed the 1 s.d. criterion). It is important for the purpose of comparing the effectiveness of the cutoffs, however, that groups of patients classified under each criterion be mutually exclusive. For example, the effectiveness of the 1.5 s.d. criterion cannot be meaningfully compared with the 2 s.d. criterion if both capture overlapping groups of subjects. Thus, once a patient was identified as PD-MCI (for example, under the 2 s.d. criterion), they were excluded from the next step in the analysis so that effectiveness of a criterion was not influenced by the more severely impaired patients who would also be captured by a more stringent criterion. That is, we began by applying the 2 s.d. criterion at baseline to all of the 121 non-dementing patients. The risk of progression to PDD was evaluated in this PD-MCI group relative to the remainder of the PD patients. This 2 s.d. PD-MCI group was then excluded and, using only the remaining patients, the risk of PDD was assessed in patients who met the 1.5 s.d. PD-MCI cutoff relative to those not meeting the 1.5 s.d. criterion. This 1.5 s.d. PD-MCI group was in turn also excluded and the remaining sample reassessed. This final sample was used to assess the risk of PDD in patients now meeting only the 1s.d. cutoff relative to the risk in the remaining patients who did not meet any of the PD-MCI criteria at baseline. Following the MDS-TF recommendations, the minimum of two impairments at the cutoff required for any given criterion could appear anywhere within, or across, the five cognitive domains assessed.

#### Part two

The second part of the analysis was different in that it examined the influence of whether the minimum of two impaired scores occurred either within one cognitive domain or across two different cognitive domains. Impairments beyond two deficits did not change these allocations. This approach was used only for the optimal cutoff score (1.5 s.d.), as determined by Part One (Figure 4, Part Two). Here, we once more began with the entire followed-up sample and again followed a stepwise approach so that each PD-MCI group consisted of a discrete (independent) sample of patients. We first identified all patients with a minimum of two impairments at 1.5 s.d. within a single cognitive domain at baseline (that is, all patients from the entire sample of 121 patients who had two or more scores at or worse than the 1.5 s.d. cutoff score) and determined the risk of dementia within this group of PD-MCI patients relative to those who did not meet this criterion. Those meeting that criterion were then discarded. The remaining patients were then assessed by comparing risk in those patients who had the minimum of two impairments at the cutoff score of 1.5 s.d. but the two impairments never occurred within a single domain (i.e., at least two domains each having a maximum of one impaired score) relative to the remaining patients who did not meet either of these two PD-MCI criteria. As any patient with multiple impairments within a single domain had been excluded in the previous stage, then by definition this latter PD-MCI group had only one impaired score within any one domain. In clinical practice, this second more relaxed 1.5 s.d. criterion would also capture those with multiple impairments within a domain, but for analysis purposes we kept the samples independent.

### Statistical analysis

Confidence intervals for relative risk of PDD in each comparison were determined using bootstrap methods.^46^ In this procedure, statistical samples were generated by resampling with replacement from the parent sample under study and a relative risk calculated. This resampling was repeated 5,000 times, resulting in an empirical distribution for the relative risk for the comparison in question. Permutation tests were used to derive exact *P* values. The code and data used in this analysis are available upon request.

## Figures and Tables

**Table 1:** Demographics and neuropsychological data at study entry (mean (s.d.))

	*Controls*	*Non-converters to PDD*	*Converted to PDD*
Sample size	36	96	25
Sex, M:F	25:11	63:33	14:11
Age	67.5 (8.6)	64.3 (8.2)	70.2 (5.7)^a^
Symptom duration (y)		5.3 (4.0)	7.8 (5.0)^a^
Hoehn and Yahr stage		1.9 (0.6)	2.5 (0.7)^a^
UPDRS (motor)		31.4 (16.2)	40.7 (15.4)^a^
Patients with hallucinations		13	13^b^
Education (y)	13.7 (2.7)	13.1 (2.7)	12.5 (2.8)
Premorbid IQ (WTAR)	112 (10.0)	111 (8.0)	109 (8.2)
CDR	0.0 (0.0)	0.2 (0.2)^c^	0.5 (0.2)^a^^,^^c^
Reisberg IADL	0.2 (0.2)	0.4 (0.4)	1.1 (0.6)^a^^,^^c^
DRS-2 (AESS)	12.8 (2.1)	11.7 (2.0)	10.7 (2.1)^c^
ADAS-Cog	5.1 (2.1)	6.6 (2.9)	10.0 (2.5)^c^
MoCA	26.9 (2.0)	26.1 (2.5)	23.6 (2.5)^a^^,^^c^
Global *Z* score	0.64 (0.42)	0.06 (0.53)^c^	−0.81 (0.47)^a^^,^^c^
1. Executive function	0.73 (0.56)	0.12 (0.71)^c^	−0.81 (0.64)^a^^,^^c^
2. Attention, working memory & processing speed	0.37 (0.55)	−0.06 (0.55)^c^	−0.77 (0.51)^a^^,^^c^
3. Episodic memory	0.95 (0.81)	0.06 (0.88)^c^	−1.05 (0.69)^a^^,^^c^
4. Visuoperceptual/ visuospatial	0.52 (0.51)	0.11 (0.60)^c^	−0.62 (0.67)^a^^,^^c^
5. Language^d^	−0.07 (0.44)	0.12 (0.45)	−0.36 (0.50)^a^

Abbreviations: ADAS-Cog, Alzheimer’s dementia assessment scale-cognitive; CDR, clinical dementia rating; DRS-2 (AESS), dementia rating scale-2 (age and education scaled score); Global *Z* score, mean derived from domains 1–4; IADL, instrumental activities of daily living; MoCA, Montreal Cognitive Assessment; PDD, patients who met Level II criteria for Parkinson disease with dementia; UPDRS (Motor), Unified Parkinson’s disease rating scale (motor component); WTAR=Wechsler test of adult reading for premorbid IQ; 1–5, mean *Z* scores in each cognitive domain.

a Significantly different from PD non-converters, Tukey *post hoc* tests, *P*<0.05.

b Significantly different proportion than PD non-converters, chi-square, *P*=0.0001.

c Significantly different to controls, Tukey *post hoc* tests, *P*<0.05.

d Eight controls and 37 PD patients did not have language measures at baseline.

**Table 2:** Conversion to PDD and reversion to non-PD-MCI status

*Criteria*	*Three primary PD-MCI criteria*			*Two alternative 1.5 s.d. PD-MCI criteria*	
	*2 s.d.*	*1.5 s.d.*	*1 s.d.*	*2 in 1 domain*	*1 in each of 2 domains*
PD-MCI (*n*/remaining sample)	46/121	10/75^a^	40/65^b^	37/121^c^	19/84^d^
Relative risk (95% CI)^e^	4.2 (2.2–10.1)	4.9 (1.4–15.1)^a^	1.9 (0.3–4.3)^b^	7.2 (3.4–16.6)^c^	1.7 (0.5–7.4)^d^
PDD conversions from PD-MCI group	18/46 (39%)	3/10 (30%)^a^	3/40 (8%)^b^	19/37 (51%)^c^	2/19 (11%)^d^
Proportion of all PDD conversions	18/25 (72%)	3/25 (12%)^a^	3/25 (12%)^b^	19/25 (76%)^c^	2/25 (8%)^d^
Sex of PDD converters (M:F)	10:8	2:1^a^	1:2^b^	10:9^c^	2:0^d^
Reverted to non-PD-MCI^f^	9/46 (20%)	1/10 (10%)^a^	8/40 (20%)^b^	1/37 (3%)^c^	3/19 (16%)^d^

Abbreviations: 95% CI, confidence interval of relative risk; non-PD-MCI, patients not meeting the PD-MCI criterion; PDD, Parkinson’s disease patients who met level II criteria for Parkinson’s disease with dementia at follow-up; PD-MCI, Parkinson’s disease patients with mild cognitive impairment at baseline.

a Additional PD-MCI patients captured by the 1.5 s.d. cutoff score, excluding the 46 who had already met the more stringent 2 s.d. cutoff.

b Additional PD-MCI patients captured by the 1 s.d. cutoff score, excluding the 56 who had already met the more stringent 2 s.d. and 1.5 s.d. cutoffs.

c PD-MCI patients, within the whole sample of 121 at baseline, who met the criterion of having two impairments at 1.5 s.d. within a single domain.

d Additional PD-MCI patients captured by this cutoff score excluding those who had already met the two impairments in one-domain criterion.

e Incidence rate in PD-MCI group divided by the incidence rate in the non-PD-MCI group, which is statistically significant when the lower bound of the CI is greater than 1.

f The number of PD patients reverting back to normal cognition rather than remaining PD-MCI or progressing to PDD, according to the PD-MCI cutoff criterion being examined.
